# Cerebral infarction in centrum semiovale presenting with hemichorea: a case report and literature review

**DOI:** 10.3389/fneur.2023.1249464

**Published:** 2023-10-30

**Authors:** Jingjing Yi, Lingru Zhang, Tao Zhang, Jianfeng Li, Yifan Zhang, Meining Zhou

**Affiliations:** ^1^Department of Neurology, The Third Affiliated Hospital of Xi’an Medical University, Xi’an, China; ^2^Key Laboratory of Shaanxi Province for Craniofacial Precision Medicine Research, College of Stomatology, Xi’an Jiaotong University, Xi’an, China; ^3^Laboratory Center of Stomatology, College of Stomatology, Xi’an Jiaotong University, Xi’an, China; ^4^Department of Pharmacy, College of Stomatology, Xi’an Jiaotong University, Xi’an, China; ^5^Department of Imaging, Shaanxi Provincial People’s Hospital, Xi’an, China

**Keywords:** hemichorea, centrum semiovale, stroke, movement disorder, chorea

## Abstract

Hemichorea caused by cerebral infarction in the centrum semiovale is a rare condition that can often be misdiagnosed. In this case report, we present a rare case of a 66-year-old man who experienced involuntary movement in his left limbs due to acute cerebral infarction in the centrum semiovale. The patient did not have any obvious inducements for the hemichorea. In this case, the treatment approach followed the guidelines for treating acute cerebral infarction, combined with the use of dopamine receptor blockers. The involuntary movements gradually improved and completely remitted after 5 days of treatment, with no relapse within the following 6 months. To summarize, this case report highlights the rare occurrence of hemichorea caused by cerebral infarction in the centrum semiovale. Prompt recognition and appropriate treatment are essential to prevent misdiagnosis and ensure optimal management of the condition.

## Introduction

Hemichorea is a spectrum of involuntary movement involving one side of the body. It usually results from a lesion in the contralateral basal ganglia structure. Hemichorea can be caused by various factors, such as infections, autoimmune diseases, drug-induced disorders, metabolic diseases, neurodegenerative diseases (1), and cerebrovascular diseases like acute cerebral infarction (2, 3). However, the incidence of hemichorea caused by acute cerebral infarction is relatively low, around 1% (4, 5). It is important to identify this condition from other potential causes of hemichorea. We present a rare case of a 66-year-old man who experienced involuntary movement in his left limbs due to acute cerebral infarction in the centrum semiovale.

## Case presentation

A 66 years old man with medical history of hypertension, type 2 diabetes and rectal cancer treatment was admitted to the hospital for experienced involuntary movement of the left side had started suddenly 10 h before. The principal manifestation of involuntary movement was uncontrollable writhe, which is persistent and worsen during activity or negative emotion.

No abnormalities were noted on general physical examination. On neurological examination revealed involuntary, irregular movements of the left limbs without speech disturbances, loss of consciousness, hallucinations, or limb weakness. There were no abnormalities of tendon reflexes or the sensory system. The National Institutes of Health Stroke Scale (NIHSS) on examination was 0.

Blood cell counts, liver and kidney function, blood fat, electrolytes, thyroid function, tumor markers, antistreptolysin “O” test, rheumatoid factor, the three items of vasculitis, serology for human immunodeficiency virus and *Treponema pallidum* were all normal. Random blood glucose was 6.8 mmol/L on admission. Fasting serum glucose level (5.6–8.5 mmol/L) and postprandial blood glucose (10.0–14.9 mmol/L) were abnormal. Glycosylated hemoglobin was 9.20%.

**Figure 1:**
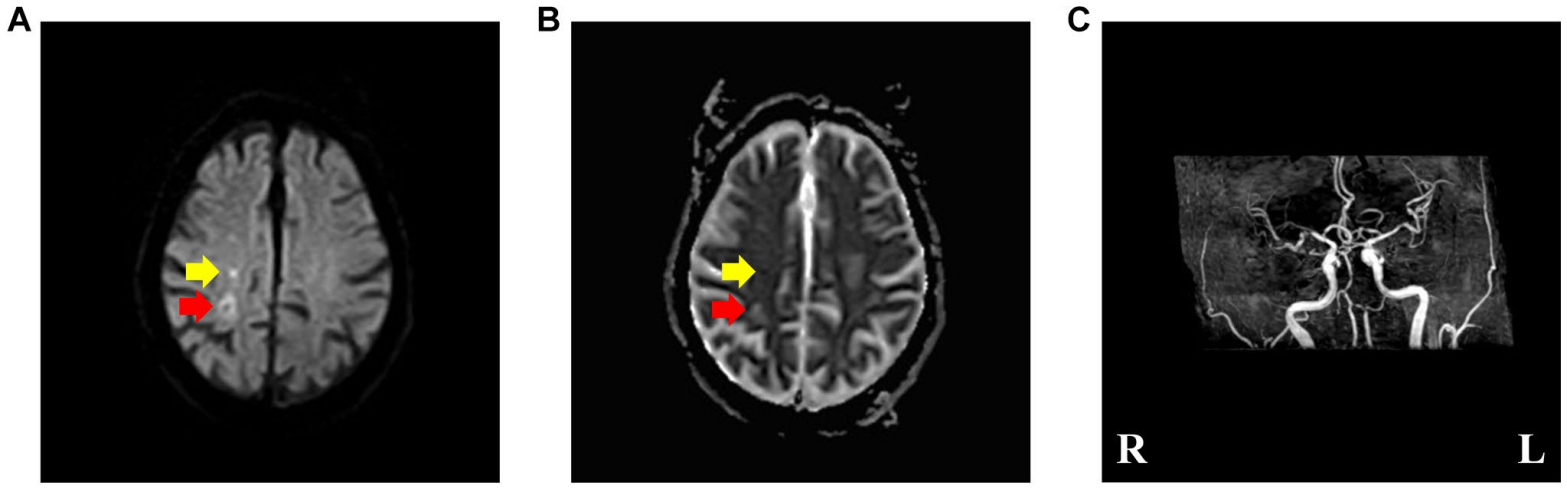
**(A,B)** Brain diffusion weighted imaging and apparent diffusion coefficient, revealing acute ischemic stroke (pointed by yellow arrow) and subacute ischemic stroke (pointed by red arrow) involving the right centrum semiovale. **(C)** Brain magnetic resonance angiography, suggesting multiple cerebral arteriosclerosis and stenosis of cerebral artery.

**Figure 2:**
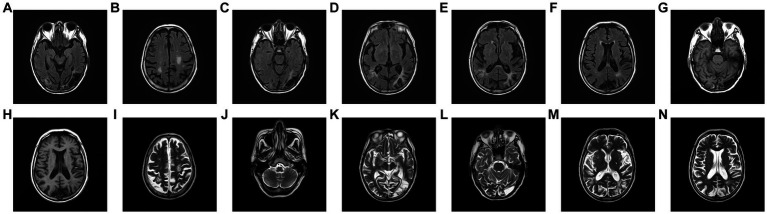
**(A–F)** Fluid-attenuated inversion recovery. **(G,H)** Brain T1-weighted image. **(H–N)** T2-weighted image, revealing multiple old infarction and encephalomalacia foci combined with gliosis of bilateral frontal, parietal, occipital and temporal lobe. Virchow-Robin Spaces and demyelination was observed in the basal ganglia of both sides, and no obvious abnormalities were observed in the brainstem.

The head diffusion weighted imaging (DWI, Figure 1) scan showed acute infarction in the right centrum semiovale. Head magnetic resonance imaging (MRI, Figure 2) results revealed multiple cerebral softening lesions with gliosis in the bilateral frontal, parietal, occipital, and temporal lobes.

Multiple cerebral arteriosclerosis and stenosis of the right middle cerebral artery were also observed from magnetic resonance angiography (MRA, Figure 1).

The acute infarction was corresponding to the timing of the hemichorea episode. The older infarct showed in the DWI, presented high signal intensity on ADC (pointed by red arrow in Figure 1), indicating that the lesion may be the lesion of subacute cerebral infarction and had nothing to do with the present attack. Therefore, the possibility of hemichorea caused by old cerebral infarction is unlikely.

**Figure 3:**
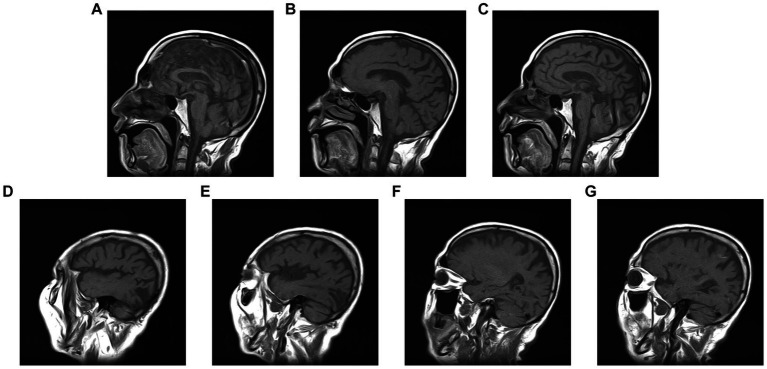
**(A–G)** No legions showed in striatum from Brain T1-weighted image sagittal images.

Although our patient had elevated fasting and postprandial blood glucose levels upon admission, the presentation did not meet the diagnostic criteria for non-ketotic hyperglycinemia (NKH). Moreover, there were no characteristic imaging findings such as high T1W1 signal in the right striatum observed on the head MRI (Figure 3). Therefore, the possibility of hemichorea caused by NKH is ruled out.

Furthermore, we noted that the patient had a history of rectal cancer surgery and underwent relevant examinations after admission. Generally, lung cancer, breast cancer, and ovarian cancer are the main causes of paraneoplastic neurological syndromes, whereas rectal cancer is relatively rare in clinical practice. Considering that rectal cancer is associated with a high risk of brain metastasis, we still considered the impact of cancer. All results showed no tumor recurrence or metastasis.

In addition, the patient denied the history of taking special drugs (such as tricyclic antidepressants, central nervous system stimulants et al.) and poisons, so drug or poison-related hemichorea was not considered. The possibility related to thyroid abnormalities and central nervous system vasculitis were also not taken into consideration due to the normal items and lack of clinical manifestations.

Taking all factors into account, we diagnosed the patient with hemichorea caused by acute cerebral infarction in the centrum semiovale.

The patient was treated with aspirin, atorvastatin, troxerutin, and haloperidol. The regiment of treatment were 5 mg haloperidol administration through intramuscular injection on the day of admission, and then intramuscularly inject 5 mg once a day until discharge. Aspirin tablet 100 mg and atorvastatin 20 mg orally once a day, and troxerutin 300 mg intravenously once a day were also given while in hospital.

The involuntary movements gradually improved from the day after the admission and completely remitted after 5 days of treatment, with no relapse within the following 6 months.

## Discussion

Cerebral infarction in the centrum semiovale typically leads to contralateral hemiparesis, sensory abnormalities, and aphasia, but rarely presents with hemichorea (6). The most common locations for hemichorea caused by stroke are the basal ganglia (caudate nucleus, putamen, globus pallidus) and subthalamic nucleus (4, 7, 8). Other reported sites of infarction include the frontal, parietal, occipital, midbrain, and pons (8–10). Hemichorea is associated with infarctions involving the territories of the middle cerebral artery and posterior cerebral artery, which supply the basal ganglia (8). Additionally, Pondal M et al. reported chorea of the lower limbs secondary to cavernoma (11).

Typically, when hemichorea accompanies small-sized cerebral infarctions, the pyramidal tracts are usually preserved. When the infarction area is large and involves the pyramidal tracts, paralysis of the contralateral limbs is usually severe, but hemichorea does not occur. However, ipsilateral chorea can occur due to compression of the contralateral brain. In our reported case, the patient only exhibited hemichorea and did not show signs of pyramidal tract involvement or limb weakness. The possible pathophysiology is as follows: (I) disruption of the neurotransmitter balance in the extrapyramidal system. Specifically, decreased activity of inhibitory neurotransmitters (gamma-aminobutyric acid) and enhanced activity of excitatory neurotransmitters (glutamate and dopamine) result in chorea and (II) Extrapyramidal circuits consist of the direct pathway (cerebral cortex – striatum – globus pallidus interna/ substantia nigra pars reticulata – ventral thalamus – cerebral cortex) and the indirect pathway (cerebral cortex – striatum – globus pallidus externa – subthalamic nucleus – globus pallidus interna – ventral thalamus – cerebral cortex) (1, 7, 9). Cerebral infarction in the centrum semiovale may damage the fibers in the cortical-basal ganglia-thalamocortical loop, leading to excessive inhibition of the indirect pathway or activation of the direct pathway, without damaging the pyramidal tracts, resulting in hemichorea as the sole clinical manifestation. Hemichorea caused by acute cerebrovascular disease typically occurs on the contralateral side while ipsilateral chorea usually occurs in patients with large cerebral infarction and intracerebral hemorrhage (7). It is worth noting that there have also been reports of generalized chorea caused by unilateral anterior cerebral artery infarction and hemichorea caused by ipsilateral subdural hematoma (12, 13).

Due to constraints, this study also has some other limitations. Although we considered the TOAST classification as being consistent with small artery occlusion, Holter monitoring and a bubble test were not performed to exclude the possibility of cardioembolic stroke due to patient preference. Additionally, the patient did not undergo a follow-up head MRI and DWI after symptom resolution, which would have been helpful in confirming our diagnosis. SPECT, electroencephalography, and cerebral perfusion imaging would have provided more persuasive evidence.

This rare case highlights the importance of considering acute cerebral infarction as a potential cause of hemichorea, even when the location is outside the basal ganglia, such as in the centrum semiovale. It’s crucial for clinicians to be aware of this possibility to avoid misdiagnosis and ensure timely treatment.

The treatment principles for hemichorea caused by acute cerebral infarction are similar to those for acute cerebral infarction itself. Antiplatelet therapy to prevent further thrombus formation and improve cerebral circulation is important. Additionally, interventions aimed at improving cerebral arteriosclerosis and circulation, such as blood pressure control, lipid management, and lifestyle modifications, should be considered. In terms of symptomatic treatment for choreic movements, dopamine receptor blockers like haloperidol can be prescribed to alleviate symptoms (14). These medications help modulate the neurotransmitter imbalance in the extrapyramidal system and reduce excessive dopaminergic activity, thereby reducing chorea (15). This combination proved to be effective in relieving the patient’s symptoms.

It’s worth noting that patients with hemichorea caused by cerebral infarction often retain good reconstruction ability of the extrapyramidal system (16). This may contribute to the relatively favorable prognosis observed in these patients. Rehabilitation therapy, including physical and occupational therapy, could be beneficial in promoting functional recovery.

Overall, increasing awareness of the association between hemichorea and acute cerebral infarction, as well as implementing appropriate diagnostic and treatment strategies, can lead to better management and outcomes for patients with this condition.

## Data availability statement

The raw data supporting the conclusions of this article will be made available by the authors, without undue reservation.

## Ethics statement

The studies involving human participants were reviewed and approved by the Ethics Committee of the Third Affiliated Hospital of Xi’an Medical University. The patients/participants provided their written informed consent to participate in this study. Written informed consent was obtained from the individual(s) for the publication of any potentially identifiable images or data included in this article.

## Author contributions

JY: collecting information and writing – original draft. LZ: diagnosing and treating. TZ: collecting information. JL and YZ: interpretation of imaging results. MZ: project administration. All authors contributed to the article and approved the submitted version.
